# Context‐dependent outcomes in a reproductive mutualism between two freshwater fish species

**DOI:** 10.1002/ece3.1979

**Published:** 2016-01-25

**Authors:** Brandon K. Peoples, Emmanuel A. Frimpong

**Affiliations:** ^1^ Department of Fish and Wildlife ConservationVirginia Polytechnic Institute and State University100 Cheatham HallBlacksburgVirginia24061

**Keywords:** Biotic interaction, context, fish, freshwater, mutualism, nest association, *Nocomis*, stream, vertebrate

## Abstract

1. The development of encompassing general models of ecology is precluded by underrepresentation of certain taxa and systems. Models predicting context‐dependent outcomes of biotic interactions have been tested using plants and bacteria, but their applicability to higher taxa is largely unknown.
2. We examined context dependency in a reproductive mutualism between two stream fish species: mound nest‐building bluehead chub *Nocomis leptocephalus* and mountain redbelly dace *Chrosomus oreas*, which often uses *N. leptocephalus* nests for spawning. We hypothesized that increased predator density and decreased substrate availability would increase the propensity of *C. oreas* to associate with *N. leptocephalus* and decrease reproductive success of both species.
3. In a large‐scale in situ experiment, we manipulated egg predator density and presence of both symbionts (biotic context), and replicated the experiment in habitats containing high‐ and low‐quality spawning substrate (abiotic context).
4. Contradictory to our first hypothesis, we observed that *C. oreas* did not spawn without its host. The interaction outcome switched from commensalistic to mutualistic with changing abiotic and biotic contexts, although the net outcome was mutualistic.
5. The results of this study yielded novel insight into how context dependency operates in vertebrate mutualisms. Although the dilution effect provided by *C. oreas* positively influenced reproductive success of *N. leptocephalus*, it was not enough to overcome both egg predation and poor spawning habitat quality. Outcomes of the interaction may be ultimately determined by associate density. Studies of context dependency in vertebrate systems require detailed knowledge of species life‐history traits.

## Introduction

A major goal in ecology is to understand context dependency in biotic interactions. Typically mutualistic interactions are particularly prone to context dependency (Chamberlain et al. 2014); they can become commensalistic or even parasitic along biotic and abiotic gradients (Karst et al. 2008), with important population‐ and community‐level ramifications (Miller et al. 2009; Bever et al. 2012). We still lack a concerted predictive framework for understanding context‐dependent mutualisms (Agrawal et al. 2007), in part because of disproportionate research among ecosystems and taxa (He and Bertness 2014). For example, context dependency in ant–plant and mycorrhizal mutualisms have been studied so thoroughly as to allow system‐specific syntheses and meta‐analyses (Chamberlain and Holland 2009; Hoeksema et al. 2010). Meanwhile, animals contributed only 16.7% of mutualism studies to a recent meta‐analysis of context dependency, and vertebrates comprised only 2.7% (Chamberlain et al. 2014). This is due largely to the fact that few studies have examined context dependency in positive interactions among vertebrates (Blanc and Walters 2008; Gingins et al. 2013; Canestrari et al. 2014; Peoples et al. 2015a). Furthermore, only a handful of studies have considered positive interactions in freshwater ecosystems (Holomuzki et al. 2010), and even fewer have investigated how those interactions may change with context (Chamberlain et al. 2014; Skelton et al. 2014). Freshwater fishes clearly provide a novel system with which to advance our general understanding of biotic interactions.

Most mutualisms involve exchange of trophic resources or protective services (Boucher et al. 1982), and most studies focus on these types of resources (Becker and Grutter 2005; Wyatt et al. 2014). Food and protection represent indirect proxies of fitness; for example, increased food resources improve reproductive capacity via body condition, increased energy to devote to reproduction, etc. However, mutualists can also exchange novel resources to facilitate reproduction (Bergmüller et al. 2007). Reproductive mutualisms provide unique opportunities for understanding context dependency because interaction outcomes (in this case, reproductive success) represent a direct proxy of fitness. Reproductive mutualisms among vertebrates may be particularly useful for understanding context dependency because they readily allow for the decisions of individuals to be evident in population performance (Bshary and Schäffer 2002; Slater et al. 2007; Carter and Wilkinson 2013). Such application may provide novel insight into the mechanisms by which biotic interactions structure animal communities.

In this study, we investigated context dependency in a novel vertebrate–vertebrate reproductive mutualism: nest associative spawning stream fishes of eastern North America. Adult male *Nocomis* (Cyprinidae) reproduce by carrying stones in their mouths to construct conspicuous gravel mounds for spawning; they bury eggs in gravel after spawning. *Nocomis* facilitates the reproduction of over 35 other cyprinid species. Collectively termed “nest associates,” these species require host nests for spawning to various degrees. Nest association behavior may be nearly obligate for some “strong” associates, but many “weak” associates can either spawn with a host or revert to the ancestral behavior of open‐substrate broadcast spawning (Johnston and Page 1992; Pendleton et al. 2012). Nest associates are lithophils (requiring gravel substrate for spawning, sensu Balon 1975); their reproductive success depends on the presence of concentrated gravel and the relative absence of silt, which can smother eggs and larvae. *Nocomis* nesting can create spawning habitat where gravel is scarce (McManamay et al. 2010) or heavily embedded with silt (Peoples et al. 2011). However, associates may not rely on hosts as heavily in habitats where high‐quality spawning substrate is abundant. Further, because associates do not guard their broods, egg burying by hosts can confer increased survival to associates that would be otherwise unattainable by spawning without a host; this constitutes a form of parental care (Johnston 1994a). Likewise, *Nocomis* can benefit from nest association through a dilution effect; high proportions of associate eggs on nests reduce the probability of host brood being eaten by egg predators. Therefore, nest associative mutualists trade two key resources: substrate and egg dilution. Nest association is context‐dependent: Among various taxa and systems, pairwise outcomes of nest association have been documented as parasitic (Fletcher 1993; Yamana et al. 2013), commensalistic (Shao 1997), and mutualistic (Goff 1984; Johnston 1994b; Peoples and Frimpong 2013).

We conducted a large‐scale in situ experiment to investigate context dependency in the nest association between *Nocomis leptocephalus* and *Chrosomus oreas*, two common cyprinids in the central Appalachian Mountains, USA. *Chrosomus oreas* is a strong associate (Pendleton et al. 2012), but can also spawn by open‐substrate broadcasting in the natural absence of a host (Jenkins and Burkhead 1994). Our objectives were to elucidate the effect of both abiotic (substrate availability) and biotic (egg predator density) context on the behavior and fitness outcomes (reproductive success) of the interaction. Substrate availability was represented by riparian land use types: forested habitats with abundant high‐quality gravel (hereafter, “control”) and deforested habitats in which riparian erosion caused all gravel to be covered with a layer of silt (hereafter, “silted”). Biotic context was represented by densities of *Etheostoma flabellare* and *Catostomus commersoni*, two egg predators common throughout the Appalachians. In a few experimental units, postnuptial male *Campostoma anomalum* (which often attempt to disrupt spawning and feed on eggs, Sabaj et al. 2000) were used as a substitute for *C. commersoni* due to shortage of the latter. For comparison of gains in fitness relative to baseline conditions, we used juvenile (larval) abundance as the proxy for fitness (sensu Chamberlain and Holland 2009).

We hypothesized that the outcomes of nest association between *N. leptocephalus* and *C. oreas* would depend on both abiotic and biotic context. We hypothesized that increased predator density (biotic context) would increase the propensity of *C. oreas* to associate with *N. leptocephalus*. The threat of egg predation should increase the perception by *C. oreas* that *N. leptocephalus* nests are safer, given the extra parental care provided by *N. leptocephalus*, relative to spawning on open substrate. Likewise, we hypothesized that reduced availability of clean gravel substrate (abiotic context) would increase the propensity of *C. oreas* to associate with *N*. *leptocephalus* by limiting potential spawning microhabitat choices for *C. oreas;* nests of *N. leptocephalus* are often the only sources of unsilted gravel substrate in degraded stream reaches. Overall, we expected high predator density and decreased substrate availability to decrease net reproductive success of both species. We also hypothesized that the effects of substrate availability and predator density on reproductive success of either symbiont species would depend on the presence of the other symbiont species (biotic context). We expected the net effect of one symbiont species on reproductive success of the other to be positive (i.e., a mutualistic relationship), but expected gross contextual outcomes to range from negative, to neutral, to positive.

## Methods

### Study site and experimental methods

We conducted this study in spring of 2012 and 2013 in three third‐ to fourth‐order streams in the Valley and Ridge physiographic province of southwestern Virginia. Each stream represented a major drainage basin: Toms Creek (2012, Gulf of Mexico), North Fork Roanoke (2012, Atlantic), and Catawba Creek (2013, Chesapeake Bay) (Table 1). We chose streams of different basins to block for potential basin‐specific effects that could confound results (e.g., different geomorphologies, discharge profiles, water chemistry). Control reaches were characterized by extensive riparian vegetation, stable banks, and relatively little silt accumulation in riffles. Conversely, silted reaches were highly entrenched, had little to no riparian vegetation, and exhibited considerable sediment accumulation in riffles. Reaches on the same stream were separated by at least 1.5 fluvial km. Placing reaches in close proximity was logistically optimal and allowed us to replicate the experiment in contrasting habitats without significant differences in stream size.

**Table 1:** Coordinates for three experimental whole plots

Stream	Habitat type	Latitude	Longitude
North Fork Roanoke River	Control	37.30	−80.26
North Fork Roanoke River	Silted	30.29	−80.27
Toms Creek	Control	37.37	−80.42
Toms Creek	Silted	37.26	−80.43
Catawba Creek	Control	37.38	−80.10
Catawba Creek	Silted	37.38	−80.09

**Figure 1:**
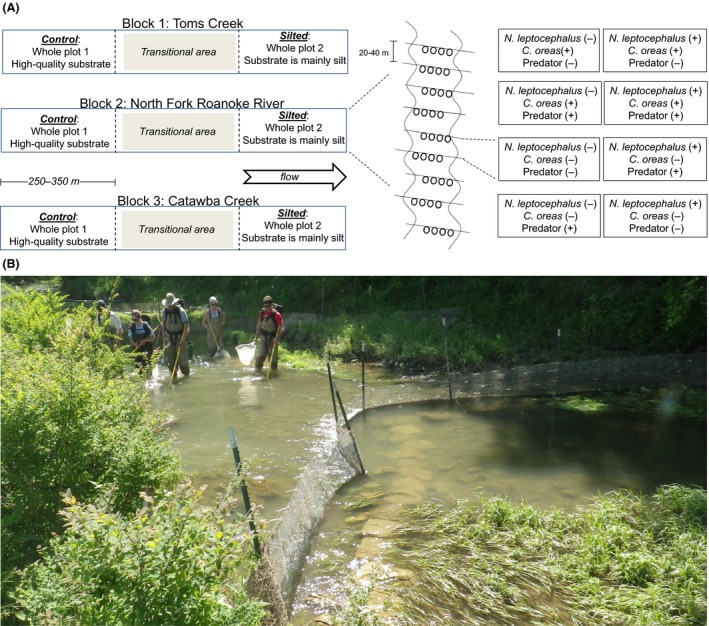
(A) Stocking densities are as follows: *N. leptocephalus* (−) = 0/unit and (+) = 16/unit; *C. oreas* (−) = 0/unit and (+) = 30/unit; predator (−) = 10/unit with only *C. oreas* or *N. leptocephalus* and (−) = 20/unit with both *C. oreas* and *N. leptocephalus*; predator (+) = 30/unit with only *C. oreas* or *N. leptocephalus* and 60/unit with both *N. leptocephalus* and *C. oreas*. There was no stocking in the (−, −, −) treatment, and the remaining treatment had 60 egg predators. Adjustment to dace and chub abundance is made to keep predation pressure constant across treatments. We set predator density to “high” and “low” instead of “present and absent” to present the most realistic conditions possible: *N. leptocephalus* and *C. oreas* sometimes occur in the absence of one another (Peoples and Frimpong 2015), but they always exist in the presence of some form of egg predator. (B) Depletion electrofishing to clear EUs of all fishes before restocking.

We conducted an *in situ* experiment of a balanced, split‐plot 2^3^ factorial design. Whole plots were replicated in control and silted habitats on each stream (*n *=* *3 whole plots per habitat type, 6 total). Each whole plot contained eight experimental units (EUs), randomly assigned a two‐level (+, −) treatment of (a) predator density, (b) *N. leptocephalus* presence, and (c) *C. oreas* presence (Fig. 1A). This provided three replicates per treatment and 48 EUs. Experimental units were instream enclosures constructed of 6.4‐mm mesh hardware cloth, supported by steel posts and backed by two‐panel strips of ~5 × 10 cm welded fencing. To secure enclosures from fish movement among EUs, we partially excavated substrate directly upstream of fences and bent ~40 cm of the bottom portion of fences upstream to form an apron. We then buried fence aprons as much as possible and secured the entire margin with 23‐kg, form‐fitting sandbags. Several fences were constructed upstream of the experiment to minimize the potential for large‐floating material and larval fishes to enter the experiment from upstream. We constructed fences in riffles to ensure each EU contained riffle habitat to provide opportunity for nonassociative spawning by *C. oreas*; pool tail habitat to provide opportunity for *N. leptocephalus* nesting; and pool habitat for feeding, resting, and cover for either species. In other words, each EU contained an entire channel geomorphic sequence, which spanned between 20 and 40 m. We constructed fences in a downstream‐facing “V” shape to reduce water pressure on enclosures (Fig. 1B). We placed the downstream‐most point of each fence (the apex of the “V”) in the thalweg of the channel (the deepest point and typically point of greatest flow). This design required daily maintenance (cleaning fences to prevent clogging and overflow), but withstood multiple large floods and required minimal postflood repair.

We removed all fishes from EUs using triple‐backpack electrofishing. We electrofished until no adult fishes (>40 mm) were captured; this required between four and ten electrofishing passes, depending on EU length and habitat complexity. During removal, we retained fish in instream, flow through holding tanks and monitored them for signs of handling stress. No fish was restocked into the experiment if it exhibited signs of handling stress (e.g., lethargy, labored breathing, erratic swimming). We then restocked fish at predetermined densities (Fig. 1) and released remaining individuals outside the experiment. Stocking densities were calculated to represent natural densities identified by previous sampling in nearby streams (Peoples and Frimpong 2011, 2013; Pritt and Frimpong 2014). We conducted spawning observations twice daily using methods described in detail by Peoples et al. (2015a). Two workers wearing polarized sunglasses, one on each side of the stream, walked the length of the experiment and located fish to record whether or not they were spawning. Spawning of *N. leptocephalus* was evidenced by the presence of a conspicuous gravel mound in the experimental unit. Spawning of *C. oreas* was evidenced by multiple individuals in breeding color congregated in swift water and schooling vigorously. We are confident that no spawning events went undetected because (a) spawning by both species is conspicuous and can last for several days and (b) because the streams used in this study are relatively small (no more than 8 m wide and 1.5 m deep). For the purposes of this study, this method of surveying for spawning activity was superior to snorkeling, which can be quite disruptive as our study species can be skittish during spawning. If spawning was occurring, we video recorded (if water clarity permitted) or observed activities for at least half an hour each time to identify behavioral interactions.

At the onset of spawning, we attached 2‐mm mesh vinyl screen to enclosures, leaving a 30‐cm gap at the most downstream end of each fence (the point of the “V,” not shown in Fig. 1B). At the gaps, we fastened 250‐micron ichthyoplankton nets to capture drifting larval fishes from each unit. Approximately three days after spawning, larval fishes entered the drift and became susceptible to capture by drift nets. Capture of larval fishes was facilitated by the V‐shaped fences, which funneled much of instream flow to the points where drift nets were located. By gently directing water across the entire fence (now covered by fine nylon mesh) using long‐handled brushes, we were able to direct all drifting material into the net at the downstream end of each EU. Drift samples were also collected upstream of the experiment to minimize fish larvae to enter the stream from outside the EU. Drift samples were collected twice daily for two weeks after the onset of spawning. Larval fishes become less susceptible to drift sampling as they become more mobile and begin schooling. Accordingly, we also collected larval fishes with cylindrical light traps designed specifically for sampling in shallow gravelly margins of small streams (approximately 10 cm in diameter by 30 in length, baited with submersible LED lights sensu Gyekis et al. 2006). We set light traps at dusk and retrieved them each morning. All larval fish samples were preserved in 90% ethanol.

### Analyses

We identified larval fishes to species using discriminant function analysis (DFA) of morphomeristic characters, corroborated by DNA bar coding. Briefly, we measured/calculated several morphometric characters on separately collected larval fish samples from North Fork and Catawba Creek in 2012 and 2013, respectively. These characters include preanal length, postanal length, eye diameter, head height, preanal and postanal myomere counts, ratios of pre‐ to postanal lengths and myomere counts, eye‐to‐head diameters, head‐to‐preanal length and head‐to‐total length (TL). Morphometric variables were log‐transformed to improve normality and subjected to DFA. Each larval fish was then identified using DNA sequencing of the mitochondrial COI locus. We edited raw DNA sequences in Sequencher, v4.5, and compared sequences against entries in GenBank using the Basic Local Alignment Search tool (Altschul et al. 1990). We then compared classification rates for DFA to known genetic identifications using cross‐validation and resubstitution procedures (Peoples, Cooper, Hallerman & Frimpong, unpubl.).

We analyzed four response variables to test predictions of the BMM, resulting in one separate model for each response variable. For each species, one response was a binary variable – whether or not spawning occurred. This variable represents choices made by individuals to either engage in exchange of reproductive resources (substrate and egg dilution) or to spawn alone (i.e., aggregated, but without the other species) and not engage in nest association. The other two response variables were the natural‐log‐transformed (to meet assumptions of approximate normality for linear models) counts of larval individuals of both species; this variable represents a direct proxy of reproductive success – the outcomes of reproductive interactions. We constructed mixed models to account for nested error structure by introducing a random factor of each whole plot nested within habitat type and included second‐order interaction terms (Potcner and Kowalski 2004). Variables in each model included predator density, habitat type, and symbiont presence (i.e., models predicting *C. leptocephalus* presence, and vice versa). Each model also contained second‐order interactions between the main factors. We fit models using maximum likelihood estimation and used contrasts to tease apart effects of specific factor levels and combinations within significant interaction effects. All analyses were conducted using SAS 9.3 (SAS Institute, Cary, NC, USA). Effects were considered significant at *α *= 0.05. However, due to modest sample size, we also interpret marginally significant effects of *P* ≤ 0.10.

## Results

A large flood on Toms Creek near the end of reproductive activity breached experimental units and washed away all nests. Analyses of spawning initiation are therefore based on data from all three systems (*n = *48 EUs), but analyses of reproductive success were only possible for Catawba Creek and North Fork Roanoke (*n = *32 EUs). Results suggest that nest association behavior is obligate for *C. oreas*, which spawned only in nine of 24 EUs in which it was stocked, and all spawning occurred in the presence of breeding *N. leptocephalus*. *C. oreas* did not initiate reproduction until male *N. leptocephalus* began nest construction. *C. oreas* did not spawn in the three remaining experimental units with *N. leptocephalus* that turned out to be reproductively inactive. *C. oreas* in EUs without *N. leptocephalus* did not enter into intense breeding color and were usually observed schooling sluggishly at medial depths in slow current; this is not spawning behavior. After experiments, female *C. oreas* from several EUs without *N. leptocephalus* were collected and dissected and were found to be full of eggs; those in units with *N. leptocephalus* contained few to no eggs. It is highly unlikely that this observation constitutes differences in maturity stage at the beginning of the experiment because all individuals were taken within the same study reach and randomly assigned to EUs, and because they were all generally the same size (within 10 mm). Analyses revealed that the only factor predicting whether or not *C. oreas* spawned was the presence of a reproductively active adult male *N. leptocephalus*. Neither habitat type nor predator density predicted whether or not *C. oreas* would spawn. Conversely, *N. leptocephalus* constructed nests in 19 of 24 EUs in which they were stocked, regardless of *C. oreas* presence. No experimental factor (i.e., habitat quality, predator density, or *C. oreas* presence) predicted whether or not *N. leptocephalus* constructed nests (Table 1).

**Figure 2:**
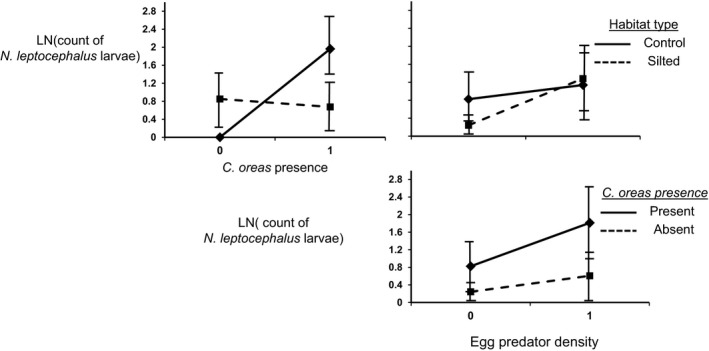
Interaction plots depicting second‐order effects of combinations of *Chrosomus oreas* presence, egg predator density, and habitat type on the abundance of larval *Nocomis leptocephalus*. Points represent natural‐log‐transformed treatment means, bounded by standard errors. The presence of *C. oreas* interacted significantly with habitat type to predict reproductive success of *N. leptocephalus* (A). Interactions between predator density and habitat type (B) and predator density and presence of *C. oreas* (C.) were not statistically significant. See Figure 1 for description of egg predator densities.

The net outcome of nest association between *N. leptocephalus* and *C. oreas* was mutualistic; *N. leptocephalus* positively affected abundance of larval *C. oreas*, and *C. oreas* moderately but also positively affected abundance of larval *N. leptocephalus*. In fact, no larval fishes were collected in units with *C. oreas* in the absence of *N. leptocephalus*. The net effect of *C. oreas* presence on *N. leptocephalus* larval abundance was relatively weak because species presence contrasted with habitat. As a main effect, habitat type did not directly affect larval abundance of either species (Table 2). However, habitat type interacted with the presence of *C. oreas* to affect abundance of larval *N. leptocephalus*. Contrasts revealed that in the absence of *C. oreas*,* N. leptocephalus* reproductive success did not differ between habitat types (*F*_1,23_ = 1.79, *P* = 0.1923). In the presence of *C. oreas,* however, the effect of habitat type on larval abundance of *N. leptocephalus* was marginally significant, being greater in control than in silted habitats (F_1,23_ = 4.12, *P* = 0.0532; Fig. 2). Thus, the outcome of the interaction switched from commensalistic in silted habitats to mutualistic in forested habitats. Predator density had no effect on reproductive success of either species (Table 2).

**Table 2:** Results (*F* statistic, *P*‐value) of mixed models predicting whether or not *Nocomis leptocephalus* or *Chrosomus oreas* initiated spawning, and the natural‐log‐transformed abundance of *N. leptocephalus* and *C. oreas* larvae in a large‐scale instream experiment conducted in three streams in southwestern Virginia, USA, in spring of 2012 and 2013. Effects significant at *P* < 0.05 are presented in bold font, and effects significant at *P* ≤ 0.10 are presented in italics. Degrees of freedom for *F* statistics were 38 for models of spawning initiation, and 23 for models of larval abundance. The independent variable, “Mutualist” represents *C. oreas* for models in which *N. leptocephalus* is the dependent variable, and vice versa. “Predator” represents either low or high densities of *Etheostoma flabellare* and *Catostomus commersoni* or postnuptial *Campostoma anomalum*. Habitat represents a condition of instream habitat representing substrate availability: either “control” or “silted.”

Dependent variable	Independent variable (Experimental factor)					
	Mutualist	Predator	Habitat	Mutualist * Predator	Mutualist * Habitat	Predator * Habitat
*N. leptocephalus spawning*	0.47, 0.4987	0.10, 0.7517	1.48, 0.2320	<0.01, 0.9447	<0.01, 0.9447	0.89, 0.3518
*C. oreas* spawning	**15.15, 0.0004**	0.20, 0.6598	0.55, 0.4622	0.18, 0.6779	0.18, 0.6779	0.18, 0.6779
LN(*N. leptocephalus*) larval abundance	*2.91, 0.1001*	1.67, 0.2089	0.17, 0.6798	0.36, 0.5564	*4.18, 0.0526*	0.48, 0.4963
LN(*C. oreas*) larval abundance	**9.29, 0.0057**	1.52, 0.2306	0.86, 0.3628	1.52, 0.2306	0.86, 0.3628	0.79, 0.3837

## Discussion

This study presents one of the first explicit tests of context dependency in vertebrate interactions to analyze a direct proxy of participant fitness. Our results demonstrate how individual decisions regarding interspecific resource trade scale up to affect population‐level processes. *Nocomis leptocephalus* reproduced more successfully in forested than in silted habitats, but only in the presence of *C. oreas*. Analyses of spawning behavior and reproductive success demonstrated that *C. oreas* benefitted from *N. leptocephalus*, regardless of context. The relationship was thus commensalistic in silted habitats where *C. oreas* did not improve reproductive success of *N. leptocephalus*, and only became mutualistic under improved habitat conditions. In other words, the beneficial dilution effect afforded to *N. leptocephalus* by *C. oreas* was evident in habitats with quality spawning substrate, but was not enough to overcome the deleterious effects of both predation (interpreted below) *and* suboptimal substrate in silted habitats. In this system, the outcome of nest associative spawning may be largely determined by symbiont density. Even at low densities (30 individuals per nest), the presence of nest associates can cause a net mutualistic interaction. However, although densities of focal species used in this experiment are similar to those found in local streams, in natural settings, *Nocomis* nests are typically swarmed with hundreds of associates representing multiple species (up to six species, not including *Nocomis*, Peoples et al. 2015a). These aggregations can create egg dilution by associates between 84% and 97% (Wallin 1992; Cashner and Bart 2010). Accordingly, it is likely that high associate densities created by multiple species can maintain a mutualistic relationship, even in unfavorable habitats. A logical next step for experimental research in this system will be to investigate associate abundance as a source of context dependency of this system.

Contrary to our hypothesis, we observed no effect of egg predator density on the reproductive success of either *N. leptocephalus* or *C. oreas*. This is surprising, given the strong effects of predation at determining the outcomes of nest associations in other systems (Baba et al. 1990; Fletcher 1993; Johnston 1994a). The most likely explanation for this is that egg predator density between high and low factor levels was not sufficiently different to generate a meaningful effect. This aids in explaining the interaction effects of habitat type and *C. oreas* presence on reproductive success of *N. leptocephalus*. If predation pressure was relatively equal among EUs (recall predator densities were “high or low,” not “present or absent”), then the dilution effect provided by *C. oreas* must operate evenly (at least statistically) across predator densities. Increasing predator densities (particularly of *C. commersoni*) in future experiments would be both stressful on fishes and logistically difficult. Future work may need to manipulate egg predator presence rather than density, although this may represent a departure from natural conditions.

The possibility also exists that we chose relatively inefficient egg predators. However, we observed all egg predator species congregating around nests and exhibiting feeding behavior on several occasions. For example, we observed several *E. flabellare* burrowing entirely through *N. leptocephalus* nests, and one nest sustained considerable damage after being raided by *C. commersoni*. Unfortunately, many egg predators of *Nocomis* nests are also nest associates that have already spawned, have yet to spawn, or are actively spawning on another nest. Parsing out the beneficial dilution effect and the deleterious effect of predation by additional nest associates was beyond the scope of this work, particularly as tertiary participants can dramatically affect the outcome of nest associative spawning (Baba et al. 1990). To perform this experiment, it was necessary that we operate with a simple two‐mutualist system. However, future experiments and theory development from this system will require a broader, community‐level context.

Another valuable insight from this study is that the simple presence of *N. leptocephalus* was not sufficient to induce spawning in *C. oreas*; a nest‐building male was required. This demonstrates that the hosts exert partner control over associates by dictating timing of reproduction. However, nest associates of *Nocomis* are capable of utilizing multiple hosts (*Campostoma* and *Semotilus* spp.), depending on several factors such as host availability, timing of reproduction, or nest characteristics. In fact, associates sometimes spawn with multiple hosts in relatively close spatial and temporal proximity (Grady and Cashner 1988; Peoples et al. 2015b). Accordingly, it is possible that partner control/choice by all participants operates to stabilize interaction outcomes through time (Bshary and Noë 2003; Kaltenpoth et al. 2014). However, further study is necessary to determine whether these outcomes represent conscious effort by hosts and/or associates, or are simply consequences of life‐history idiosyncrasies that vary through time and across the species' distributional ranges.

Increased sample size would have improved our statistical power and clarified marginally significant effects. Unfortunately, the timing and brevity of the cyprinid reproductive season are not conducive to large‐scale experimentation due to spring floods; most instream experiments in temperate streams are conducted during months of low flow (Angermeier and Karr 1984; Power et al. 1985; Charlebois and Lamberti 1996). However, other well‐established experimental studies of stream organisms have made contributions with comparable sample sizes (Harvey 1991; Nakano et al. 1999). Although the effects of some mechanisms may be unclear, this study provides a step forward for understanding context dependency in mutualisms among vertebrates.

We intend this study to stimulate discussion on context dependency in interactions among vertebrates. Some results contradict our hypotheses, illustrating the lack of a general understanding about interspecific vertebrate symbioses. Very few studies have sought to empirically test the degree to which reproductive symbioses are facultative in fishes (Wallin 1992; Mattingly and Black 2013). A better understanding of the basic reproductive ecology of fishes is necessary for future theoretical research in this system of interactions; experiments with truly facultative associates will yield better insight into context dependency in mutualisms among fishes. A gradient describing the strength of this trait among potential associates in the study area was previously identified (Pendleton et al. 2012) and could provide candidate alternate species for future studies improving on the design of the current experiment. Lastly, future research on other positive biotic interactions in stream fish communities, such as mixed species schooling (Matthews 1998, chapter 9), seed transport (Horn et al. 2011) and bioturbation (Flecker 1996; Moore 2006), will contribute to a better understanding of the roles played by positive interactions in animal communities.

## Conflict of Interest

None declared.
